# When is diminishment a form of enhancement? Rethinking the enhancement debate in biomedical ethics

**DOI:** 10.3389/fnsys.2014.00012

**Published:** 2014-02-04

**Authors:** Brian D. Earp, Anders Sandberg, Guy Kahane, Julian Savulescu

**Affiliations:** ^1^Faculty of Philosophy, Oxford Uehiro Centre for Practical Ethics, University of OxfordOxford, UK; ^2^Faculty of Philosophy, Institute for Science and Ethics, University of OxfordOxford, UK; ^3^Faculty of Philosophy, Oxford Centre for Neuroethics, University of OxfordOxford, UK; ^4^Faculty of Philosophy, Future of Humanity Institute, University of OxfordOxford, UK

**Keywords:** enhancement, neuroenhancement, welfare, well-being, neuroethics, bioethics, diminishment, empathy

## Abstract

The enhancement debate in neuroscience and biomedical ethics tends to focus on the *augmentation* of certain capacities or functions: memory, learning, attention, and the like. Typically, the point of contention is whether these augmentative enhancements should be considered permissible for individuals with no particular “medical” disadvantage along any of the dimensions of interest. Less frequently addressed in the literature, however, is the fact that sometimes the *diminishment* of a capacity or function, under the right set of circumstances, could plausibly contribute to an individual's overall well-being: more is not always better, and sometimes less is more. Such cases may be especially likely, we suggest, when trade-offs in our modern environment have shifted since the environment of evolutionary adaptation. In this article, we introduce the notion of “diminishment as enhancement” and go on to defend a *welfarist* conception of enhancement. We show how this conception resolves a number of definitional ambiguities in the enhancement literature, and we suggest that it can provide a useful framework for thinking about the use of emerging neurotechnologies to promote human flourishing.

## Introduction

Advances in neuroscience and related fields have allowed for an unprecedented increase in our ability to intervene in brain-level processes, thereby influencing a wide range of higher-order functions and behaviors. As Nagel (2010) has noted, this growing and ever more finely-tuned capacity to tamper with even normally-functioning neural systems raises a number of ethical questions about the boundary between traditional research/clinical practice and outright human enhancement. “[A] societal climate of performance measurements and improvements,” Nagel writes, has led to a “growing tendency to use medical and technological means beyond their applications in classical therapy” (p. 1). Hence, even though “most of these technologies are developed for medical or research purposes, their application for [human] enhancement interventions is at hand” (*ibid.*).

A burgeoning academic literature has begun to debate the moral propriety of such enhancement. This debate often centers on the use of neurotechnological, pharmacological, or other interventions to *increase* some human capacity or function (e.g., Bostrom, 2003, 2009; Bostrom and Roache, 2008; see also Daniels, 2000; Harris, 2007; and Kass, 2003a; especially pp. 12–13). What, after all, is the meaning of “enhance” if not to heighten, to augment, to intensify? Thus we see articles asking whether non-invasive brain stimulation should be used to enhance learning (e.g., Cohen Kadosh et al., 2012); whether we should be worried about university students taking Ritalin to improve focus (e.g., Outram, 2010); whether doctors have an obligation to ingest ergogenic drugs to stay awake during late-night surgery (e.g., Greely et al., 2008); and whether the use of mood brighteners is getting out of hand (e.g., Farah, 2002). The “augmentative” flavor of much of this work can be seen in a recent article by Dees (2007, p. 372):

[Now or in the near future] drugs may be able to improve our ability to think. Amphetamines can help people to learn skilled motor tasks, like playing the piano, more rapidly. Cholinsterase inhibitors now help [patients] to improve their attention and memory, and better versions may help virtually anyone. Amphetamines, like Ritalin, improve focus, attention, and memory… Some drugs may help the formation of long-term memories and thereby facilitate learning… [Drugs can also] alter people's moods…. Soon drugs almost certainly will be developed that will “brighten” the mood of anyone who takes them.

Bostrom and Roache (2008) take a similar approach: “One important way in which the human condition could be changed is through the enhancement of basic human capacities… There are various ways in which we can currently improve [these capacities, including] stamina, strength, dexterity, flexibility, coordination, agility, and conditioning” (pages 1 and 7^1^). In another paper, Bostrom (2009, p. 8^2^) asks us to

Consider, for example, enhancements in executive function and self-control, concentration, or of our ability to cope with stressful situations; further, consider enhancements of mental energy that would make us more capable of independent initiative and that would reduce our reliance on external stimuli such as television; consider perhaps also enhancement of our ability to withstand mild pains and discomforts, and to more effectively self-regulate our consumption of food, exercise, and sleep.

Note the specific focus here on *capacities, moods*, or *functions* that might be improved by the pharmacological (or other) intervention—“improved” in the sense of facilitating *more* of whatever it is that the function normally does (see Dresler et al., 2013 for a recent review). We can summarize this sort of approach as follows:

**The Functional-Augmentative Approach to Enhancement**: Interventions are considered enhancements insofar as they improve some capacity or function (such as cognition, vision, hearing, alertness) by increasing the ability of the function to do what it normally does.

The debate then typically turns on whether the proposed capacity-enhancement should be considered *permissible* for someone who does not suffer a “medical” disadvantage along that dimension (Daniels, 2000; Allhoff et al., 2009). In this context, a distinction is frequently drawn between “enhancement” (on the one hand) and mere “treatment” or “therapy” (on the other), with the implication often being that the former may be morally problematic in ways that the latter may not be. This consideration suggests a second approach to understanding enhancement:

**The Not-Medicine Approach to Enhancement (Treatment vs. Enhancement)** (see, e.g., Sabin and Daniels, 1994; Juengst, 1998; Daniels, 2000; Kass, 2003b; Pellegrino, 2004): On this view, “the term *enhancement* [characterizes] interventions designed to improve human form or functioning *beyond what is necessary to sustain or restore good health*” (Juengst, 1998, emphasis added).

There are other approaches as well. In an earlier work, we identified two additional ways of understanding enhancement—the sociological-pragmatic approach and the ideological approach (see Savulescu et al., 2011, for details)—and then proceeded to outline a new account of enhancement, which we argued was preferable to the others: the welfarist approach. This approach can be defined as follows:

**The Welfarist Approach to Enhancement:** “Enhancement” should be defined to mean any change in the biology or psychology of a person which increases the chances of leading a good life in a given set of circumstances.

In the present article, we wish to develop our defense of this welfarist account by contrasting it specifically with the “augmentative” functionalist approach^3^ (i.e., enhancement of some capacity; see Table 1 for a selective summary)—as well as the related “not-medicine” approach to enhancement—in light of recent discussions in neuroethics that seem to break the conventional mold. We introduce the notion of “diminishment as enhancement” and focus on a set of cases in which “subtractive” interventions—that is, interventions geared toward *weakening* a given capacity or function—might plausibly contribute to individual welfare enhancement in line with the definition just laid out. We conclude by discussing some of the advantages that this welfarist conception has over other common definitions.

**Table 1:** **Selective summary of “augmentative” neural enhancements, means, and references**.

**Function**	**Means**	**References**
Attention	Nicotine, Modafinil, caffeine, glucose, aerobic exercise, rTMS, computer training, meditation	Benton et al., 1994; Hilgetag et al., 2001; Rezvani and Levin, 2001; Newhouse et al., 2004; Repantis et al., 2010; Smith et al., 2010; Chiesa et al., 2011; Zelinski et al., 2011
Empathy and mind-reading	Oxytocin, MDMA	Bartz et al., 2010; Hysek et al., 2012
Executive function	Aerobic exercise; computer training; meditation	Smith et al., 2010; Chiesa et al., 2011; Nouchi et al., 2012
Learning—including implicit learning, verbal learning, and numerical learning	Amphetamine, methylphenidate, a large number of synaptic-plasticity affecting drugs, tDCS, various memory arts and mnemonic systems	Soetens et al., 1993; Clark et al., 1999; Kincses et al., 2004; Williams and Eskandar, 2006; Lee and Silva, 2009; Cohen Kadosh et al., 2010; Repantis et al., 2010; Javadi et al., 2012; Suthana et al., 2012
Inhibitory control and self control	Modafinil, Atomoxetine, glucose	Turner et al., 2003; Galliot et al., 2007; Chamberlain et al., 2009
Memory—including working memory, memory encoding, and memory consolidation	Glucose, donepezil, physiostigmine, exercise, tDCS, Ampakines, Modafinil, methylphenidate, computer training, protein, meditation	Elliott and Sahakian, 1997; Furey et al., 2000; Lynch, 2002; Turner et al., 2003; Barch, 2004; Marshall et al., 2004; Fregni et al., 2005; Luber et al., 2007; Jaeggi et al., 2008; Ohn et al., 2008; Riby et al., 2008; Thorell et al., 2009; Smith et al., 2010; Chiesa et al., 2011; Teo et al., 2011; Jones et al., 2012
Planning	Methylphenidate	Elliott and Sahakian, 1997
Reaction speed	Glucose	Owens and Benton, 1994
Recall	tDCS	Gagnon et al., 2010; Ross et al., 2010
Wakefulness/alertness	Caffeine, Modafinil, amphetamine, other stimulants	Hartmann and Cravens, 1976; Smith, 2002; Baranski et al., 2004

## Discussion

What do we mean by “diminishment”? We can dispense with a potential red herring. We do *not* mean to draw attention to specific neural pathways or low-level mechanisms whose weakening or disruption might go on to yield some higher-order functional outcome. For instance, stimulants such as Ritalin, sometimes used to augment focus and concentration, could in principle be understood as “diminishments” since—on at least at one level of description—they actually *limit* the reuptake of neurotransmitters, ultimately producing their stimulating effects^4^. Likewise, TMS and other forms of brain stimulation may involve *disrupting* activity in one region as a means to enhancing function in another (or at another level of description). For example, TMS can reduce interference between similar-sounding words in phonological memory (likely by disrupting the phonological store), thereby improving verbal recall (Kirschen et al., 2006). In these sorts of cases, it is the higher-order function itself that we take to be the target of enhancement (i.e., the capacity for focus, concentration, or recollection), whereas the lower-order “diminishment” is merely instrumental.

By contrast, we intend to highlight interventions that serve to diminish the higher-order capacities themselves. That is, we want to focus on interventions that make concentration (for example) *worse*—by virtue of *whatever* neural mechanism is involved. Consider some illustrative cases. Should soldiers be given propranolol to *reduce* the emotional intensity of wartime memories (e.g., Henry et al., 2007)? Should a battered spouse use “anti-love” neurotechnology to *sever* the emotional attachment she has with her abuser (Earp et al., 2013)? Should sex offenders have to undergo “chemical *castration*” as a condition of parole (e.g., Gupta, 2012)? Should appetite *suppressants* be developed for mass-market consumption (e.g., Farah, 2013)? These are just a few recent examples of potential interventions that might reduce or diminish a higher-order capacity.

Interventions of this kind raise many of the same patterns of ethical concern as the more conventional cases of functional enhancement typically encountered in the bioethics literature. For example: who should administer the drug or apply the technology? Should the intervention be regulated? How? Is a threat to autonomy or authenticity potentially implied? And what sort of externalities might need to be anticipated?

At the same time, however—given a functional-augmentative framework—these cases might seem puzzling or out of place. They seem puzzling because they apparently involve the very *opposite* of enhancement, namely, diminishment: i.e., *diminishment* of wartime memories; *diminishment* of harmful love; *diminishment* of ill-directed lust, and so on. How might these seemingly opposite-to-enhancement outcomes be made to square with the seemingly similar-to-enhancement applicability of “standard” bioethical analysis?

There is a straightforward solution to this puzzle. Sometimes, diminishment *is* enhancement—on the welfarist definition of the term (see above). That is, once we shift our focus from the particular *capacity* or *function* being modified to the overall *normative goal* of the modification itself, we begin to see that “enhancement” may be more broadly understood as having something to do with well-being—a goal that the welfarist definition makes explicit. On this account, in order for an intervention to count as an enhancement, it does not matter if the capacity itself is being modified “up” or being modified “down.” Nor does it matter if the modification is being accomplished by means^5^ of a drug, a biochip, an electrical brain-stimulator, or something more familiar and lower tech. Nor does it matter if the intervention is called “medicine” or “therapy” or “beyond therapy” or anything else. If it increases the person's chances of leading a good life in the relevant circumstances, then we propose that it should be considered an enhancement.

### Implications of the argument

Well… so what? What does this welfarist definition get us? How will it be *useful* for medical professionals, neuroethicists, and other stakeholders engaged in these sorts of discussions? Finally, what advantages does it have over other definitions used throughout the literature?

First, and most basically, it acknowledges that “more” is not always “better.” As commonplace a maxim as this is, it is not always duly appreciated. As the neuroscientist Baron-Cohen (2011) has recently argued, even such “obviously” beneficial human capacities as the ability to empathize may have mal-adaptive consequences in certain cases. For example, too much empathy might drive a person to prioritize attending to others' feelings over meeting her own basic needs. Or consider “empathy fatigue”—a term used by Stebnicki (2007) to refer to the physical and emotional exhaustion that grief and trauma counselors sometimes come to face: their inability to *distance* themselves emotionally from the pain and suffering of their clients ultimately prevents them from doing their job. Likewise, Williams (1989) has hypothesized that among helping professionals, high emotional empathizers may be disposed to earlier career burnout.

The same lesson may apply to other “obviously” beneficial capacities such as intelligence or IQ. While super-intelligence might seem to be an enviable trait or disposition, being “too smart for one's own good” is not always a mere teasing admonition: for many intellectually gifted individuals, very high intelligence can come at a direct cost to their overall well-being (Harrison and Van Haneghan, 2011). Furthermore, intelligence is not sufficient for achieving the good life, but is merely instrumental, and IQ enhancement *per se* does not seem to have any clearly determinate value (Tännsjö, 2009).

Likewise, the ability to remember well would seem to be a beneficial thing: “memory makes us” (it has been said) and many elderly people are very sad to see their powers of recollection fade over time. But as any victim of rape might tell you—and as the soldiers we referred to earlier would hasten to agree—sometimes memory can be a devastating shackle. In addition, research has shown that excessive autobiographical memory (hyperthymesia) can interfere with the basic business of living one's life (Parker et al., 2006).

Finally, even romantic love—undoubtedly the most celebrated emotional capacity of all—can be dangerous or even life-threatening when the object of affection is cruel or abusive. Some victims of domestic violence, for example, find themselves unable to diminish their passionate feelings for their abuser, despite being fully aware that their long-term well-being and even basic physical safety may be under threat by remaining in the relationship (Earp et al., 2013, 2014, under review; see also Earp et al., 2012, for further discussion).

The implication in all of this is clear; and here we reaffirm our thesis to drive it home: Sometimes, diminishing a certain *capacity* or *function*—under the right set of circumstances—could quite plausibly *enhance* a person's overall well-being.^6^

### Some fine-tuning

Given this possibility, one might be tempted to argue that functional diminishment would only enhance well-being by bringing the individual back from a *pathological* state to a species-typical state. Yet while this direction of change could reasonably describe a number of specific cases, it is unlikely to hold as a general rule. This is because what it is that enhances well-being is not species-general, but rather context-specific. Thus, as Dees (2007) notes, “beta blockers [can be used to] decrease stress and nervousness, and so they help even normal people cope with abnormal situations” (p. 372). In the context of a public performance, for example, even a quite ordinary stress reaction could interfere with an individual's well-being, given the context-local goals of the performer: hence “[the] widespread use [of beta-blockers] among concert performers is legendary” (*ibid.*) Likewise, in the notorious “Ashley case” (Liao et al., 2007), the parents of a severely brain-impaired child wanted to stunt her growth by using estrogen therapy (as well as remove her uterus and breast buds) in order to improve her quality of life. While parts of the treatment and even the motivations behind it may certainly be called into question, it is at least plausible to think that reducing Ashley's growth, all things considered, would count in favor of her own best interests. For example, Ashley's parents suggested that her smaller size would make it easier to carry her around, thus allowing her to participate more fully in the activities of daily living. If so, then approaching the normal human size range would *not* improve well-being, whereas the proposed diminishment might.

But what is the more general thrust of the argument? In other words, when, or under what conditions, is (functional) diminishment likely to produce (well-being) enhancement? We have already discussed context-specific and “pathological” cases, but others suggest themselves as well. One plausible view is that we should expect promising enhancers when the trade-offs in our living conditions have shifted from the environment of evolutionary adaptation (Bostrom and Sandberg, 2008). While some changes no doubt enable well-being enhancement in the sense of getting *more* of something that was limited in the past because of its cost, there are likely other domains in which something has lost importance today. For example, fight-or-flight reactions that would have served well in an environment flush with predators might today contribute more to stress, cardiovascular disease, and problems with anxiety (Bracha and Maser, 2008). Anti-parasite immune cells can become overactive in our relatively clean environment, triggering allergies (Sironi and Clerici, 2010). And ancient hunger drives can lead to obesity in today's societies, given the unprecedented availability of low-nutrient, high-calorie foods (e.g., Serlie et al., 2011). These examples provide further potential cases in which we might judiciously *diminish* our body's natural responses in order to improve our overall well-being.

## Conclusion

Our aim in this article has been simple. It has been to rethink, or at least to problematize, the chiefly “augmentative” flavor of discourse surrounding neurotechnological enhancement. We do not mean to imply, of course, that all or even most of the diminishments we have discussed are currently technologically feasible, nor do we suggest that they would always be the best solution to the problem at hand. As Levy (2012) has recently argued, when faced with a detrimental mismatch between our capacities and our context, it is often better to change our environmental conditions than it is to re-tool our biology, all things considered. Other times, the best course of action might be to pursue a complementary strategy that involves the use of brain-level interventions alongside other types of approaches (Savulescu and Sandberg, 2008). The answer is likely to be different for different cases.

Yet whatever position one takes on the proper balance of intervention strategies, we have tried to show that there is something to be gained by distinguishing functional enhancement from enhancement of well-being. For any disposition, trait, or function, there is likely to be a discrete range of optimal levels (of intensity, sensitivity, etc.) for the given set of conditions, and too much or too little may detract from health or happiness. Identifying diminishment as a possible form of human enhancement, therefore, invites us to ask whether we may have *too much* X for the best life, based on the relevant local circumstances and other facets of modern living.

Another advantage of the welfarist definition of enhancement is that it can handle not only cases of functional diminishment (our emphasis in this paper), but even unusual cases including *extensions* of the body, in which a new capacity is added that did not exist before. One example of such a case is the use of implanted magnets for “magnetic vision” (Larratt, 2004). While this type of intervention is clearly “augmentative” (in the sense of adding something rather than subtracting), it differs from the usual augmentative cases—which involve intensifying an existing capacity—in that it introduces a novel capacity that would not exist at all without the enhancement. Similarly other forms of body-modifications may be seen as attempts to enhance well-being through bringing the body more in line with personal ideals of self-expression. These cases, too, may not involve the “enhancement” of any existing capacity, but rather welfare enhancement more broadly construed through the employment of biotechnology.

We are careful to note that we have left untouched important questions about who would administer these new technologies, under what specific conditions; how their advisability would be decided upon (especially as compared to other, potentially less invasive, forms of intervention); how long their effects should be expected to last; what risks or side-effects might be involved; and whether any regulatory structures would have to be put in place to accommodate their existence. These types of questions are bound to overlap with analogous puzzles being worked out in the “augmentative” enhancement literature, so we will leave their discussion for another day. Here we have endeavored, not to cultivate a vast procedural forest, but rather to plant a conceptual seed.

### Final thoughts

As we have argued elsewhere (Savulescu et al., 2011), the “enhancement debates” in biomedical ethics have been needlessly encumbered by the existence of a hodge-podge of ill-defined, poorly articulated notions of enhancement—often only implicitly communicated—along with endless to-ing and fro-ing about the relationship between enhancement and the limits of medicine. Re-casting “enhancement” as being essentially related to welfare, however, provides several distinct advantages:

It ties enhancement to the value of well-being… It offers a general framework for thinking about well-being. It offers more than a mere list of value claims. It singles out well-being as one dimension of value that is constitutive of genuine human enhancement. But it leaves open substantive and contentions questions about the nature of well-being, and important empirical questions about the impact of some treatment on well-being. [Moreover], the welfarist approach distinguishes ways in which some treatment might benefit a person from other relevant values, such as justice. It thus allows us to say that although some treatment is an enhancement (i.e., contributes to individuals' well-being), it might nevertheless be bad overall, because its employment in the current social context will lead to far greater injustice. (p. 7)

Finally, we note that people's normal brain functions will differ across time and circumstance. They will differ between people as well. We can now *control* function, at least in part, through the use of neurotechnology and biomedicine, and our ability to do so is likely to become increasingly more potent as well as more targeted in the decades to come. We suggest that we should tie this ability to a robust account of well-being (e.g., Kahane and Savulescu, 2008; Earp et al., under review), and seek to maximize function toward that end, whether the capacity itself is being augmented (as is typically emphasized in these debates) or indeed (as we emphasize here) effectively diminished.

## Authors' contributions

Brian D. Earp wrote the first draft of the essay, and undertook final edits to synthesize the contributions of the other authors. Anders Sandberg provided some of the scientific examples, and drafted the argument about shifting evolutionary trade-offs. Guy Kahane contributed substantial intellectual content and edited and approved the final draft. Julian Savulescu conceived the general argument and contributed substantially to the concluding paragraphs.

## Authors' information

Brian D. Earp studied philosophy, psychology, and cognitive science at Yale and Oxford universities and is a Research Fellow in the Oxford Uehiro Centre for Practical Ethics as well as a Consultant with the Oxford Centre for Neuroethics. Anders Sandberg trained in computer science, neuroscience, and medical engineering, and is a James Martin Research Fellow at Oxford's Future of Humanity Institute. Guy Kahane holds two degrees in philosophy from the University of Oxford and serves as Deputy Director of both the Uehiro Centre for Practical ethics and the Oxford Centre for Neuroethics. Julian Savulescu is qualified in medicine, bioethics, and analytic philosophy, and holds the Chair in Practical Ethics at the University of Oxford.

### Conflict of interest statement

The authors declare that the research was conducted in the absence of any commercial or financial relationships that could be construed as a potential conflict of interest.

## Footnotes

^1^Page numbers are from the online version available at http://www.nickbostrom.com/ethics/human-enhancement.pdf

^2^Page number is from the online version available at http://www.nickbostrom.com/ethics/dignity-enhancement.pdf

^3^One may wonder just how common this approach is—that is, how frequently it is encountered in the literature compared to other approaches. As noted above, in addition to the work of prominent figures such as Bostrom—who define enhancement explicitly in terms of augmentation of functions or capacities (see especially Bostrom, 2009)—the most commonly referenced account is the “not-medicine” approach, which distinguishes enhancement from treatment, as in the seminal report from the President's Council on Bioethics, “Beyond Therapy” (Kass, 2003b). “Therapy” is ordinarily understood as being an attempt to address disease, which on the dominant account after Boorse (1977) is species-typical subfunctioning (see also Daniels, 1985). Therefore, on the “not-medicine” approach as well, enhancement just is the improvement of some function within (or beyond) the normal range. We do not suggest, of course, that the functional-augmentative view is the only view one encounters in the literature, nor that cases of functional diminishment are never employed in these debates. Indeed, one of the classic examples of “enhancement” from the field—the blunting of painful memories (which we discuss below)—is quite common. However, in our reading of the literature, it is examples relating to capacity augmentation that are much more frequently encountered and actually used as illustrations of enhancement; and when instances of diminishment are raised, their specific implications for the conceptual understanding of enhancement is rarely if ever addressed. In addition, the link between the intervention (whether augmentative or diminishing) and *well-being* is not commonly articulated as such, with other goals such as “self improvement” being either stated or implicitly assumed (e.g., Farah, 2013).

^4^Though note, as a reviewer points out: diminishment-as-enhancement “can work at both lower (neurobiological) and higher (mental, psychological) levels in a complementary way [depending upon the way the enhancement is described]. While methylphenidate produces its stimulating effect by limiting the reuptake of dopamine, it enhances one's capacity to be more attentive to and focused on a particular task by diminishing the content and scope of one's attention” (emphasis added). Thus we can see that it is possible to augment (via diminishment) a given capacity (here, focus), not only by interfering with some lower-order neurological process, but even by diminishing an inversely related higher-order capacity: in this example, the scope of one's attention. Therefore it is important to make clear (as we do in a subsequent paragraph) that our emphasis in this paper is on interventions that would diminish the targeted higher-order capacity itself (i.e., focus), rather than either (a) some lower-order mechanism whose diminishment would actually increase the target capacity, or (b) an inversely-related higher-order capacity, whose diminishment would have a similar effect.

^5^Note that the means “do not matter” only in the specific sense stated—i.e., in terms of whether some intervention should be counted as an enhancement. By contrast, the means might very well matter in terms of having different safety profiles, etc. Furthermore, there is no reason to think that all means will be normatively equivalent either, even if well-being is taken as the explicit goal of enhancement. This is because there are other normatively-relevant considerations besides well-being, such as justice or fairness, which may impact upon the evaluation of means (as well as other factors related to enhancement), as we discuss in a later section.

^6^This sort of welfare enhancement is likeliest to occur, we suggest, when the functional diminishment in question results in optimal levels of capacity functioning for successful adaptation to the demands of the environment.

## References

[B1] Allhoff F. Lin P. Moor J. Weckert J. (2009). Ethics of human enhancement: 25 questions & answers. Stud. Ethics Law Technol. 4, 1–50 10.2202/1941-6008.1110

[B2] Baranski J. V. Pigeau R. Dinich P. Jacobs I. (2004). Effects of modafinil on cognitive and meta−cognitive performance. Hum. Psychopharmacol. Clin. Exp. 19, 323–332 10.1002/hup.596 15252824

[B3] Barch D. (2004). Pharmacological manipulation of human working memory. Psychopharmacology 174, 126–135 10.1007/s00213-003-1732-3 15205883

[B4] Baron-Cohen S. (2011). Autism, empathizing-systemizing (e-s) theory, and pathological altruism, in Pathological Altruism, eds Oakley B. Knafo A. Madhaven G. Wilson D. S. (Oxford: Oxford University Press), 344–348

[B5] Bartz J. A. Zaki J. Bolger N. Hollander E. Ludwig N. N. Kolevzon A. (2010). Oxytocin selectively improves empathic accuracy. Psychol. Sci. 21, 1426–1428 10.1177/0956797610383439 20855907 PMC6634294

[B6] Benton D. Owens D. S. Parker P. Y. (1994). Blood glucose influences memory and attention in young adults. Neuropsychologia 32, 595–607 10.1016/0028-3932(94)90147-3 8084417

[B7] Boorse C. (1977). Health as a theoretical concept. Philos. Sci. 44, 542–573 10.1086/288768

[B88] Bostrom N. (2003). Human genetic enhancements: a transhumanist perspective. J. Value Inq. 37, 493–506 10.1023/B:INQU.0000019037.67783.d5 17340768

[B8] Bostrom N. (2009). Dignity and enhancement. Contemp. Read. Law Soc. Justice 2, 84–115

[B9] Bostrom N. Roache R. (2008). Ethical issues in human enhancement, in New Waves in Applied Ethics, ed Ryberg J., (New York, NY: Palgrave Macmillan), 120–152

[B10] Bostrom N. Sandberg A. (2008). The wisdom of nature: an evolutionary heuristic for human enhancement, in Human Enhancement, eds Savulescu J. Bostrom N. (Oxford: Oxford University Press), 375–416

[B11] Bracha H. S. Maser J. D. (2008). Anxiety and posttraumatic stress disorder in the context of human brain evolution: a role for theory in DSM−V? Clin. Psychol. Sci. Pract. 15, 91–97 10.1111/j.1468-2850.2008.00113.x

[B12] Chamberlain S. R. Hampshire A. Müller U. Rubia K. Del Campo N. Craig K. (2009). Atomoxetine modulates right inferior frontal activation during inhibitory control: a pharmacological functional magnetic resonance imaging study. Biol. Psychiatry 65, 550–555 10.1016/j.biopsych.2008.10.014 19026407

[B14] Chiesa A. Calati R. Serretti A. (2011). Does mindfulness training improve cognitive abilities? A systematic review of neuropsychological findings. Clin. Psychol. Rev. 31, 449–464 10.1016/j.cpr.2010.11.003 21183265

[B15] Clark K. B. Naritoku D. K. Smith D. C. Browning R. A. Jensen R. A. (1999). Enhanced recognition memory following vagus nerve stimulation in human subjects. Nat. Neurosci. 2, 94–98 10.1038/4600 10195186

[B16] Cohen Kadosh R. Levy N. O'Shea J. Shea N. Savulescu J. (2012). The neuroethics of non-invasive brain stimulation. Curr. Biol. 22, R108–R111 10.1016/j.cub.2012.01.013 22361141 PMC4347660

[B17] Cohen Kadosh R. Soskic S. Iuculano T. Kanai R. Walsh V. (2010). Modulating neuronal activity produces specific and long-lasting changes in numerical competence. Curr. Biol. 20, 2016–2020 10.1016/j.cub.2010.10.007 21055945 PMC2990865

[B18] Daniels N. (1985). Just Health Care. Cambridge: Cambridge University Press 10.1017/CBO9780511624971

[B19] Daniels N. (2000). Normal functioning and the treatment-enhancement distinction. Camb. Q. Healthc. Ethics 9, 309–322 10.1017/S0963180100903037 10858880

[B20] Dees R. H. (2007). Better brains, better selves? The ethics of neuroenhancements. Kennedy Inst. Ethics J. 17, 371–395 10.1353/ken.2008.0001 18363271

[B21] Dresler M. Sandberg A. Ohla K. Bublitz C. Trenado C. Mroczko-Wasowicz A. (2013). Non-pharmacological cognitive enhancement. Neuropharmacology 64, 529–543 10.1016/j.neuropharm.2012.07.002 22828638

[B22] Earp B. D. Sandberg A. Savulescu J. (2012). Natural selection, childrearing, and the ethics of marriage (and divorce): building a case for the neuroenhancement of human relationships. Philos. Technol. 25, 561–587 10.1007/s13347-012-0081-8 23226627 PMC3510696

[B23] Earp B. D. Sandberg A. Savulescu J. (2014). Brave new love: the threat of high-tech “conversion” therapy and the bio-oppression of sexual minorities. Am. J. Bioeth. Neurosci. 5, 4–12 10.1080/21507740.2013.863242 PMC3932804 24587962

[B25] Earp B. D. Wudarczyk O. A. Sandberg A. Savulescu J. (2013). If I could just stop loving you: anti-love biotechnology and the ethics of a chemical breakup. Am. J. Bioeth. 13, 3–17 10.1080/15265161.2013.839752 24161170 PMC3898540

[B26] Elliott R. Sahakian B. J. (1997). Effects of methylphenidate on spatial working memory and planning in healthy young adults. Psychopharmacology 131, 196–206 10.1007/s002130050284 9201809

[B27] Farah M. J. (2002). Emerging ethical issues in neuroscience. Nat. Neurosci. 5, 1123 10.1038/nn1102-1123 12404006

[B28] Farah M. J. (2013). Cognitive Enhancement. Available online at: http://www.neuroethics.upenn.edu/index.php/penn-neuroethics-briefing/cognitive-enhancement

[B29] Fregni F. Boggio P. S. Nitsche M. Bermpohl F. Antal A. Feredoes E. (2005). Anodal transcranial direct current stimulation of prefrontal cortex enhances working memory. Exp. Brain Res. 166, 23–30 10.1007/s00221-005-2334-6 15999258

[B30] Furey M. L. Pietrini P. Haxby J. V. (2000). Cholinergic enhancement and increased selectivity of perceptual processing during working memory. Science 290, 2315–2319 10.1126/science.290.5500.2315 11125148

[B31] Gagnon G. Blanchet S. Grondin S. Schneider C. (2010). Paired-pulse transcranial magnetic stimulation over the dorsolateral prefrontal cortex interferes with episodic encoding and retrieval for both verbal and non-verbal materials. Brain Res. 1344, 148–158 10.1016/j.brainres.2010.04.041 20462501

[B32] Galliot M. T. Baumeister R. F. DeWall C. N. Maner J. K. Plant E. A. Tice D. M. (2007). Self-control relies on glucose as a limited energy source: willpower is more than a metaphor. J. Pers. Soc. Psychol. 92, 325–336 10.1037/0022-3514.92.2.325 17279852

[B33] Greely H. Sahakian B. Harris J. Kessler R. C. Gazzaniga M. Campbell P. (2008). Towards responsible use of cognitive-enhancing drugs by the healthy. Nature 456, 702–705 10.1038/456702a 19060880

[B34] Gupta K. (2012). Protecting sexual diversity: rethinking the use of neurotechnological interventions to alter sexuality. AJOB Neurosci. 3, 24–28 10.1080/21507740.2012.694391

[B35] Harris J. (2007). Enhancing Evolution: The Ethical Case for Making Better People. Princeton: Princeton University Press

[B36] Harrison G. E. Van Haneghan J. P. (2011). The gifted and the shadow of the night: Dabrowski's overexcitabilities and their correlation to insomnia, death anxiety, and fear of the unknown. J. Educ. Gift. 34, 669–697 10.1177/016235321103400407

[B37] Hartmann E. Cravens J. (1976). Sleep: effects of d-and l-amphetamine in man and in rat. Psychopharmacology 50, 171–175 10.1007/BF00430488 826958

[B38] Henry M. Fishman J. R. Youngner S. J. (2007). Propranolol and the prevention of post-traumatic stress disorder: is it wrong to erase the “sting” of bad memories? Am. J. Bioeth. 7, 12–20 10.1080/15265160701518474 17849331

[B39] Hilgetag C. C. Theoret H. Pascual-Leone A. (2001). Enhanced visual spatial attention ipsilateral to rTMS-induced ‘virtual lessons’ of human parietal cortex. Nat. Neurosci. 4, 953–957 10.1038/nn0901-953 11528429

[B40] Hysek C. M. Domes G. Liechti M. E. (2012). MDMA enhances “mind reading” of positive emotions and impairs “mind reading” of negative emotions. Psychopharmacology 222, 293–302 10.1007/s00213-012-2645-9 22277989

[B41] Jaeggi S. M. Buschkuehl M. Jonides J. Perrig W. J. (2008). Improving fluid intelligence with training on working memory. Proc. Natl. Acad. Sci. U.S.A. 105, 6829–6833 10.1073/pnas.0801268105 18443283 PMC2383929

[B42] Javadi A. H. Cheng P. Walsh V. (2012). Short duration transcranial direct current stimulation (tDCS) modulates verbal memory. Brain Stimul. 5, 468–474 10.1016/j.brs.2011.08.003 21962975

[B43] Jones E. K. Sünram-Lea S. I. Wesnes K. A. (2012). Acute ingestion of different macronutrients differentially enhances aspects of memory and attention in healthy young adults. Biol. Psychol. 89, 477–486 10.1016/j.biopsycho.2011.12.017 22223097

[B44] Juengst E. T. (1998). What does *enhancement* mean? in Enhancing Human Traits: Ethical and Social Implications, ed Parents E. (Washington, DC: Georgetown University Press), 29–47

[B45] Kahane G. Savulescu J. (2008). The welfarist account of disability, in Disability and Disadvantage, eds Cureton A. Brownlee K. (Oxford: Oxford University Press), 14–53

[B46] Kass L. R. (2003a). Ageless bodies, happy souls. New Atlantis 1, 9–28 Available online at: http://www.thenewatlantis.com/publications/ageless-bodies-happy-souls 15584192

[B47] Kass L. R. (2003b). Beyond Therapy: Biotechnology and The Pursuit of Happiness. Washington, DC: President's Council on Bioethics

[B48] Kincses T. Z. Antal A. Nitsche M. A. Bártfai O. Paulus W. (2004). Facilitation of probabilistic classification learning by transcranial direct current stimulation of the prefrontal cortex in the human. Neuropsychologia 42, 113–117 10.1016/S0028-3932(03)00124-6 14615081

[B49] Kirschen M. Davis-Ratner M. Jerde T. E. Schraedley-Desmond P. Desmond J. (2006). Enhancement of phonological memory following transcranial magnetic stimulation (TMS). Behav. Neurol. 17, 187–194 10.1155/2006/469132 17148839 PMC5471529

[B50] Larratt S. (2004). The Gift of Magnetic Vision. BMEzine.com. Available online at: http://news.bme.com/2004/02/06/the-gift-of-magnetic-vision-the-publishers-ring/

[B51] Lee Y. S. Silva A. J. (2009). The molecular and cellular biology of enhanced cognition. Nat. Rev. Neurosci. 10, 126–140 10.1038/nrn2572 19153576 PMC2664745

[B52] Levy N. (2012). Ecological engineering: reshaping our environments to achieve our goals. Philos. Technol. 25, 589–604 10.1007/s13347-012-0065-8 23396750 PMC3566833

[B53] Liao S. M. Savulescu J. Sheehan M. (2007). The Ashley treatment: best interests, convenience, and parental decision-making. Hastings Cent. Rep. 37, 16–20 10.1353/hcr.2007.0027 17474341

[B54] Luber B. Kinnunen L. H. Rakitin B. C. Ellsasser R. Stern Y. Lisanby S. H. (2007). Facilitation of performance in a working memory task with rTMS stimulation of the precuneus: frequency-and time-dependent effects. Brain Res. 1128, 120–129 10.1016/j.brainres.2006.10.011 17113573

[B89] Lynch G. (2002). Memory enhancement: the search for mechanism-based drugs. Nat. Neurosci. 5, 1035–1038 10.1038/nn935 12403980

[B55] Marshall L. Mölle M. Hallschmid M. Born J. (2004). Transcranial direct current stimulation during sleep improves declarative memory. J. Neurosci. 24, 9985–9992 10.1523/JNEUROSCI.2725-04.2004 15525784 PMC6730231

[B57] Nagel S. K. (2010). Too much of a good thing? Enhancement and the burden of self-determination. Neuroethics 3, 109–119 10.1007/s12152-010-9072-6

[B58] Newhouse P. A. Potter P. Singh A. (2004). Effects of nicotinic stimulation on cognitive performance. Curr. Opin. Pharmacol. 4, 36–46 10.1016/j.coph.2003.11.001 15018837

[B59] Nouchi R. Taki Y. Takeuchi H. Hashizume H. Akitsuki Y. Shigemune Y. (2012). Brain training game improves executive functions and processing speed in the elderly: a randomized controlled trial. PLoS ONE 7:e29676 10.1371/journal.pone.0029676 22253758 PMC3256163

[B60] Ohn S. H. Park C. I. Yoo W. K. Ko M. H. Choi K. P. Kim G. M. (2008). Time-dependent effect of transcranial direct current stimulation on the enhancement of working memory. Neuroreport 19, 43–47 10.1097/WNR.0b013e3282f2adfd 18281890

[B61] Outram S. M. (2010). The use of methylphenidate among students: the future of enhancement? J. Med. Ethics 36, 198–202 10.1136/jme.2009.034421 20338928

[B62] Owens D. S. Benton D. (1994). The impact of raising blood glucose on reaction times. Neuropsychobiology 30, 106–113 10.1159/000119146 7800156

[B64] Parker E. S. Cahill L. McGaugh J. L. (2006). A case of unusual autobiographical remembering. Neurocase 12, 35–49 10.1080/13554790500473680 16517514

[B65] Pellegrino E. D. (2004). Biotechnology, Human Enhancement, and the Ends of Medicine. The Center for Bioethics and Human Dignity. Available online at: http://www.cbhd.org/resources/biotech/pellegrino

[B66] Repantis D. Schlattmann P. Laisney O. Heuser I. (2010). Modafinil and methylphenidate for neuroenhancement in healthy individuals: a systematic review. Pharmacol. Res. 62, 187–206 10.1016/j.phrs.2010.04.002 20416377

[B67] Rezvani A. H. Levin E. D. (2001). Cognitive effects of nicotine. Biol. Psychiatry 49, 258–267 10.1016/S0006-3223(00)01094-5 11230877

[B68] Riby L. M. McLaughlin J. Riby D. M. Graham C. (2008). Lifestyle, glucose regulation and the cognitive effects of glucose load in middle-aged adults. Br. J. Nutr. 100, 1128–1134 10.1017/S0007114508971324 18377687

[B69] Ross L. A. McCoy D. Wolk D. A. Coslett H. Olson I. R. (2010). Improved proper name recall by electrical stimulation of the anterior temporal lobes. Neuropsychologia 48, 3671–3674 10.1016/j.neuropsychologia.2010.07.024 20659489

[B70] Sabin J. E. Daniels N. (1994). Determining “medical necessity” in mental health practice. Hastings Cent. Rep. 24, 5–13 10.2307/3563458 7860291

[B71] Savulescu J. Sandberg A. (2008). Neuroenhancement of love and marriage: the chemicals between us. Neuroethics 1, 31–44 10.1007/s12152-007-9002-4

[B72] Savulescu J. Sandberg A. Kahane G. (2011). Well-being and enhancement, in Enhancing Human Capacities, eds Savulescu J. ter Meulen R. Kahane G. (Oxford: Wiley-Blackwell), 3–18

[B73] Serlie M. J. La Fleur S. E. Fliers E. Pijl H. (2011). Obesity: is evolution to blame? Neth. J. Med. 69, 156–158 Available online at: http://www.njmonline.nl/abstract.php?i=137&PHPSESSID= 21527801

[B74] Sironi M. Clerici M. (2010). The hygiene hypothesis: an evolutionary perspective. Microbes Infect. 12, 421–427 10.1016/j.micinf.2010.02.002 20178858

[B75] Smith A. (2002). Effects of caffeine on human behavior. Food Chem. Toxicol. 40, 1243–1255 10.1016/S0278-6915(02)00096-0 12204388

[B76] Smith P. J. Blumenthal J. A. Hoffman B. M. Cooper H. Strauman T. A. Welsh-Bohmer K. (2010). Aerobic exercise and neurocognitive performance: a meta-analytic review of randomized controlled trials. Psychosom. Med. 72, 239–252 10.1097/PSY.0b013e3181d14633 20223924 PMC2897704

[B77] Soetens E. Hoodge R. D. Hueting J. E. (1993). Amphetamine enhances human-memory consolidation. Neurosci. Lett. 161, 9–12 10.1016/0304-3940(93)90127-7 8255556

[B78] Stebnicki M. (2007). Empathy fatigue: healing the mind, body, and spirit of professional counselors. Am. J. Psychiatr. Rehabil. 10, 317–338 10.1080/15487760701680570

[B79] Suthana N. Haneef Z. Stern J. Mukamel R. Behnke E. Knowlton B. (2012). Memory enhancement and deep-brain stimulation of the entorhinal area. N. Engl. J. Med. 366, 502–510 10.1056/NEJMoa1107212 22316444 PMC3447081

[B80] Tännsjö T. (2009). Ought we to enhance our cognitive capacities? Bioethics 23, 421–432 10.1111/j.1467-8519.2008.00721.x 19222440

[B81] Teo F. Hoy K. E. Daskalakis Z. J. Fitzgerald P. B. (2011). Investigating the role of current strength in tDCS modulation of working memory performance in healthy controls. Front. Psychiatry 2:45 10.3389/fpsyt.2011.00045 21811474 PMC3141358

[B82] Thorell L. B. Lindqvist S. Bergman Nutley S. Bohlin G. Klingberg T. (2009). Training and transfer effects of executive functions in preschool children. Dev. Sci. 12, 106–113 10.1111/j.1467-7687.2008.00745.x 19120418

[B83] Turner D. C. Robbins T. W. Clark L. Aron A. R. Dowson J. Sahakian B. J. (2003). Cognitive enhancing effects of modafinil in healthy volunteers. Psychopharmacology 165, 260–269 10.1007/s00213-002-1250-8 12417966

[B84] Williams C. (1989). Empathy and burnout in male and female helping professionals. Res. Nurs. Health 12, 169–178 10.1002/nur.4770120307 2727323

[B85] Williams Z. M. Eskandar E. N. (2006). Selective enhancement of associative learning by microstimulation of the anterior caudate. Nat. Neurosci. 9, 562–568 10.1038/nn1662 16501567

[B87] Zelinski E. M. Spina L. M. Yaffe K. Ruff R. Kennison R. F. Mahncke H. W. (2011). Improvement in memory with plasticity-based adaptive cognitive training: results of the 3-month follow-up. J. Am. Geriatr. Soc. 59, 258–265 10.1111/j.1532-5415.2010.03277.x 21314646

